# Recommendations for the packaging and containerizing of bioinformatics software

**DOI:** 10.12688/f1000research.15140.2

**Published:** 2019-03-20

**Authors:** Bjorn Gruening, Olivier Sallou, Pablo Moreno, Felipe da Veiga Leprevost, Hervé Ménager, Dan Søndergaard, Hannes Röst, Timo Sachsenberg, Brian O'Connor, Fábio Madeira, Victoria Dominguez Del Angel, Michael R. Crusoe, Susheel Varma, Daniel Blankenberg, Rafael C. Jimenez, BioContainers Community, Yasset Perez-Riverol

**Affiliations:** 1 Bioinformatics Group, Department of Computer Science, University of Freiburg, Freiburg, 79110, Germany; 2 Institut de Recherche en Informatique et Systèmes Aléatoires (IRISA/INRIA) - GenOuest Platform, Université de Rennes, Rennes, France; 3 EMBL European Bioinformatics Institute, Cambridge, UK; 4 Department of Pathology, University of Michigan, Ann Arbor, Michigan, USA; 5 Center of Bioinformatics, Biostatistics and Integrative Biology, Institut Pasteur, Paris, France; 6 Bioinformatics Research Centre, Aarhus University, Aarhus, DK-8000, Denmark; 7 The Donnelly Centre, University of Toronto, Toronto, Ontario, M5S 3E1, Canada; 8 Applied Bioinformatics Group, Wilhelm Schickard Institut für Informatik, Universität Tübingen, Tübingen, D-72076, Germany; 9 Computational Genomics Lab, UC Santa Cruz Genomics Institute, University of California, Santa Cruz, Santa Cruz, California, USA; 10 Institut Français de Bioinformatique (Elixir-FR), UMS3601-CNRS, Université Paris-Saclay, Orsay, 91403, France; 11 Microbiology and Molecular Genetics, Michigan State University, East Lansing, Michigan, USA; 12 Genomic Medicine Institute, Lerner Research Institute, Cleveland Clinic, Cleveland, Ohio, USA; 13 ELIXIR Hub, Cambridge, CB10 1SD, UK

**Keywords:** containers and packages, best practices bioinformatics, reproducibility

## Abstract

Software Containers are changing the way scientists and researchers develop, deploy and exchange scientific software. They allow labs of all sizes to easily install bioinformatics software, maintain multiple versions of the same software and combine tools into powerful analysis pipelines. However, containers and software packages should be produced under certain rules and standards in order to be reusable, compatible and easy to integrate into pipelines and analysis workflows. Here, we presented a set of recommendations developed by the BioContainers Community to produce standardized bioinformatics packages and containers. These recommendations provide practical guidelines to make bioinformatics software more discoverable, reusable and transparent.  They are aimed to guide developers, organisations, journals and funders to increase the quality and sustainability of research software.

## Introduction

The ability to reproduce the results of a scientific study is a cornerstone of the scientific method, and yet remains one of the most significant challenges in modern science. Evidence from multiple authors suggest that reproducibility in biomedical research is lower than 85% ^1^, with 90% of researchers asserting a reproducibility crisis within science ^2^. One major obstacle to reproducible science is the accurate and complete reporting of all experimental and computational steps required to obtain the described results. The ability to accurately reproduce bioinformatics analysis, including data handling and statistical downstream processing frequently poses significant challenges—even when performed by the original authors of a study ^3, 4^.

In addition, reproducing computational analysis by other researchers requires the deployment of the bioinformatic software at a different site. Previous publications have highlighted the importance of openness and availability of tools, software, scripts and data ^3, 5^, and focused on three central premises for reproducible bioinformatics software deployment: (i) documenting the version of all software, (ii) open source availability of the source code and all custom software, (iii) adopting a license and complying with third-party dependency licenses ^3, 6^.

However, even if source code and data are published in a public repository as Supplementary material to a paper, the source code may have non-obvious dependencies on other software, configuration options, operating systems and other subtleties that hamper re-usability ^7^. Building, installing, and deploying scientific software often requires additional information missing in the published manuscript or the accompanying documentation. Additionally, workflows and pipelines commonly combine software developed by different teams and groups, adding another layer of complexity and introducing challenges such as compatibility and management of dependencies, running serial and parallel processes and working with a broad variety of software types and user-defined parameters. Software containers have emerged as a powerful technology to address primary dependency issues and enable distributing and deploying scientific software in a runnable state ^8^.

Software packages are collections of computer programs along with metadata, such as dependencies, descriptions, and versions, required for distribution and deployment. Package management systems are used to search for software, resolve dependencies, and then install the requisite dependencies and software. The most common package managers exist at the level of the operating system, such as yum, apt, and pacman. These allow for the installation of software on a system-wide basis. Containers constitute lightweight software components and libraries that can be quickly packaged, are designed to run anywhere ^8^, and are useful and essential tools to leverage bioinformatics software reproducibility. Package managers are often used to create the execution environment within containers. Conda packages and Docker/Singularity containers are well-known technologies that have already gained traction in the field of bioinformatics ^9^. By May 2018, the BioContainers ^8^, and Bioconda ^10^ communities have released more than 4000 public containers, facilitating the development of complex and reproducible workflows and pipelines ^11, 12^.

This manuscript describes a core set of recommendations and guidelines to improve the quality and sustainability of research software based on software packages and containers. It provides easy-to-implement recommendations that encourage the adoption of packaging (e.g. Conda) and container (e.g. Docker, Singularity) technologies in bioinformatics and software development for research. It provides recommendations about making research software and its source code more reproducible, deployable, reusable, transparent and more compatible with other tools and software. In this manuscript, software is broadly defined to include command line tools, graphical user interfaces, application program interfaces (APIs), infrastructure scripts and software packages (e.g. R packages).

## Recommendations

### 1. A package first

A software package is self-contained software including all the dependency libraries and packages necessary to execute the software. Some of the most popular and well-known package management systems are operating system-level, such as apt or yum, are language-specific resources, such as pip/PyPI, CPAN, or CRAN, or are third-party package managers such as Zero Install, Homebrew, and Conda. When choosing a package manager, it is important to select one that is cross-platform (e.g. works on various Linux distributions and MacOS), allows multiple versions of each package, does not require administrative nor elevated privileges to use and, ideally, is not restricted to a single programming language. Being cross-platform enhances reusability, providing for multiple versions enables reproducibility, and allowing user-based installation and multiple development languages simplifies usability.

Conda, is a popular package manager in research software, it quickly installs, runs and updates packages and their dependencies. It handles dependencies for many languages, such as C, C++, R, Java, Perl, and Python. It works cross-platform and does not require special permissions for installation of itself or requested packages. Conda supports the creation of individual and unique execution environments and allows multiple versions of packages to be installed in a user-declared fashion.

The field of bioinformatics has developed an active community around Bioconda ^10^. Bioconda is a channel for the Conda package manager specialised in bioinformatics software. You can create a *Conda* package by defining a *BioConda* recipe ( Box 1). This recipe includes enough information about the dependencies, the license and fundamental metadata to find, retrieve and use the package. When a recipe is added to Bioconda, it is automatically built into a usable package, tested and made available as a Docker container via Quay.io and as a Singularity container ^13^, and is displayed in the BioContainers registry ^8^.

Box 1. **Bioconda recipe for “deepTools”, a set of user-friendly tools for normalisation and visualisation of deep-sequencing data**

package:

  name: deeptools

  version: '3.0.2'

source:

  fn: deepTools-3.0.2.tar.gz

  url:

https://files.pythonhosted.org/packages/21/63/095615a9338c824dcc1496a302d04267c674175f0081e1ee2f897f33539f/deepTools-3.0.2.tar.gz

  md5: 4553d9c828ba4b5b93ca387917649281

build:

  number: 0

requirements:

  build:

   - python

   - setuptools

   - gcc

  run:

   - python

   - pybigwig >=0.2.3

   - numpy >=1.9.0

   - scipy >=0.17.0

   - matplotlib >=2.1.1

   - pysam >=0.14.0

   - py2bit >=0.2.0

   - plotly >=1.9.0

   - pandas

test:

  imports:

   - deeptools

  commands:

   - bamCompare –version

about:

  home: https://github.com/fidelram/deepTools

  license: GPL3

  summary: A set of user-friendly tools for normalisation and visualisation of deep-sequencing data

extra:

  identifiers:

   - biotools:deeptools

   - doi: 10.1093/nar/gkw257

### 2. One tool, one container

Microservice and modular architectures ^14^ provide a way of breaking large software projects down into smaller, independent and loosely coupled modules. These software applications can be viewed as a suite of independently deployable, small, modular components in which each tool runs a unique process and communicates through a well-defined, lightweight mechanism to serve a specific task ^14^. Each of these independent modules is referred to as a *container*. A container is essentially an encapsulated and immutable version of an application, coupled with the bare-minimum operating system components (e.g. dependencies) required for execution ^8^.

Containers should be defined to be as granular as possible, with the premise of one tool, one container. Each container should encapsulate only one piece of software that performs a unique task with a well-defined goal (e.g. sequence aligner, mass spectra identification). This recommendation, one tool, one container, should be implemented carefully, keeping containers as modular and scoped in functionality as possible. Developers may use their judgement to compose a layered container based on other containerised tools. Here, we strongly recommend that the modular composition of these tools should also be exposed as a single modular tool - still abiding by “one tool, one container”.

Is important to highlight that workflow composition is not addressed in the scope of this article as we are tied to the one container = one tool paradigm. Workflow environments such as Nextflow and Galaxy will execute 1 (task, process) per tool, each tool being one container.

### 3. Tool and container versions should be explicit

The tool or software wrapped inside the container should be fixed explicitly to a defined version through the mechanism available by the package manager used ( Box 2). The version used for this main software should be included in both the metadata of the container (for ease of identification) and the container tag. The tag and metadata of the container should also include a versioning number for the container itself, meaning that the tag could look like a *version of the tool* or *version of the container*. The container version, which does not track the tool changes but the container revision, should follow semantic versioning to signal its backward compatibility.

Box 2. **BioContainers recipe (Dockerfile) for Comet software**

The public Dockerfile is here:

https://github.com/BioContainers/containers/blob/5bec8724bf61d696523216244264db11eba137d6/comet/2015025/Dockerfile

FROM biocontainers/biocontainers:v1.0.0_cv4

LABEL base_image="biocontainers:v1.0.0_cv4"

LABEL version="3"

LABEL software="Comet"

LABEL software.version="2016012"

LABEL about.summary="an open source tandem mass spectrometry sequence database search tool"

LABEL about.home=" http://comet-ms.sourceforge.net"

LABEL about.documentation=" http://comet-ms.sourceforge.net/parameters/parameters_2016010"

LABEL about.license_file=" http://comet-ms.sourceforge.net"

LABEL about.license="SPDX:Apache-2.0"

LABEL extra.identifiers.biotools="comet"

LABEL about.tags="Proteomics"

LABEL maintainer="Felipe da Veiga Leprevost < felipe@leprevost.com.br>"

USER biodocker

RUN ZIP=comet_binaries_2016012.zip && wget https://github.com/BioDocker/software-archive/releases/download/Comet/$ZIP-O/tmp/$ZIP&&unzip/tmp/$ZIP-d/home/biodocker/bin/Comet/&&chmod-R 755/home/biodocker/bin/Comet/*&&rm/tmp/$ZIP

RUN mv/home/biodocker/bin/Comet/comet_binaries_2016012/comet.2016012.linux.exe/home/biodocker/bin/Comet/comet

ENV PATH /home/biodocker/bin/Comet:$PATH

WORKDIR /data/

If a copy is done via *git clone* or equivalent, a specific commit or a tagged git version should be specified, never a branch only. Cloning a branch (master, develop, etc.) will always use the latest source code in that branch making impossible to reproduce the build process since the different source code will be built as soon as the branch is updated by the software authors. Upstream authors should be asked to create a stable version of their software with reasonable guarantees that the specified version works as advertise including passing all automated tests ( Recommendation 7)—this will often be a release version. Any patches added on top of the officially released source code should be highlighted.

For projects that practice agile software development (including continuous integration) where each version is stable, tested and works as advertised, the SVN or git identifier can be used as the tool version for the container—possibly with the addition of a date in YYYYMMDD format to easily identify newer versions from older versions. Please note that depending on the used source code management system (git, hg, svn, cvs) it is possible to remove entire commits or rewrite the commit history of a project. Therefore, the safest way is to use a release *tarball* and can be archived separately, as Bioconda and BioContainers are doing.

### 4. Avoid using ENTRYPOINT

It is a well-known feature of Docker that the entry point of the container can be over-written by definition (e.g., ENTRYPOINT [ /bin/ping]). The ENTRYPOINT specifies a command that will always be executed when the container starts. Even when the ENTRYPOINT helps the user to get a *default* behaviour for a tool, it is generally not recommended because of reproducibility concerns of the implicit hidden execution point. By explicitly executing the tool by its executable inside the container (using the container as an environment and not as a fat binary merely through its ENTRYPOINT) the user (e.g. workflow) can recognise and trace the tool that is used within the container.

***4.1. Relevant tools and software should be executable in the PATH.*** If for some reason the container needs to expose more than a single executable or script (for instance, EMBOSS or OpenMS ^11^ or other packages with many executables), these should always be executable and be available in the container’s default PATH. This will, almost always, be the case by default for everything installed via package managers (dpkg, yum, pip, etc.), but if you are adding tailored made scripts or installing by source, take care to add the executables to the PATH. This allows the container to be used as an environment or to specify alternative commands to the main ENTRYPOINT easily ( Recommendation 4).

### 5. Reduce the size of your container as much as possible

As containers are frequently pushed and pulled (uploaded and downloaded) to/from container registries over the internet, their size matters. There are multiple ways to reduce the size of your container during builds, the most efficient way is to have two different containers: one for building the app and the second container for deploying the app (which will be the one user will download). While you may need multiple libraries, source code and dependencies for building the app, you should only include the bare necessities in the deployment container, which will actually run the app. Some general guidelines that can be followed are listed below (see Supplementary File 1):

- Avoid installing "recommended" packages in apt based systems in your deployed container.
- Do not keep build tools in the deployed image (this includes compilers and development libraries). You can install these tools in the build image.
- Use a lightweight base image for your deployed container. Only use a more mainstream image such as Ubuntu or CentOS if absolutely required for your application to run. The BioContainers community provides also a set of images based on Ubuntu (see example biocontainers:v1.0.0_cv4) and Debian.

### 6. Keep data outside of the container

Data can dramatically increase the size of the container ( Recommendation 5), thereby reducing the capability to share, deploy and deposit it in public registries. In order to implement tests during the building and deployment steps, we recommend downloading or cloning the data from public data repositories and deleting it after the testing is finished. This mechanism is similar to the one stated in Recommendation 5 for retrieving source and binaries.

Many bioinformatics tools require access to large reference datasets to perform meaningful analysis. These reference datasets should also not be included within the container but should be stored in a user-configurable location and retrieved either on-demand during runtime, or as part of a setup process. Not only does storing reference datasets outside of the container reduce the size of the container, but other tools that require access to the same reference data will be able to directly access the data without additional overhead. It is also recommended that datasets themselves are versioned and all downloaded files are verified using secure cryptographic hashes.

### 7. Add functional testing logic

If others want to build your container locally, want to rebuild it later on with an updated base image, want to integrate it to a continuous integration system or for many other reasons, users might want to test that the built container still serves the function for which it was initially intended. For this, it is useful to add some functional testing logic to the container (in the form of a bash script for instance) in a standard location (here we propose a file called “runTest.sh”, executable and in the path), which includes all the logic for:

- Installing any packages that might be needed for testing, such as *wget* for instance to retrieve example files for the run.
- Obtaining sample files for testing, which might be for instance an example data set from a reference archive.
- Running the software that the container wraps with that data to produce an output inside the container.
- Comparing the generated output and exit with an error code if the comparison is not successful.

The file containing testing logic is not meant to be executed during container build time, so the retrieved data and packages do not increase the size of the container when it is built. However, because the testing file is inside the container, any user who has built the container or downloaded the container image can check that the container is working as intended by the author by executing “runTest.sh” inside the container.

### 8. Check the license of the software

When adding software or data in a container, always check the license of the resource being added. A free-to-use license is not always free to distribute or copy. The license must always be explicitly defined in your Docker labels. For some licenses, the license file needs to be shipped within the container and with the software. If a license is not specified, you should ask the upstream author to provide a license ^5, 15^.

### 9. Make your package or container discoverable

Biomedical research and bioinformatics demand more efforts to make bioinformatics software and data more findable (discoverable), accessible, interoperable, and reusable (FAIR principles) ^16^. Leveraging those principles, we recommend to the bioinformatics community and software developers to make their containers and packages more findable. To make your package available, we recommend the following steps:

- Annotate packages and containers with metadata that allows users (e.g. biologists and bioinformaticians) to find them.
- Make packages and containers available. We recommend developers to make the recipe of how to build a container available for others, including i) the source code or binaries of the original tools; ii) the configuration settings and test data.
- Register packages and container in existing bioinformatics registries helping users and services to find them.
- Registries such as BioContainers ^8^ and bio.tools ^17^ collaborate with each other by exchanging metadata and information using different APIs and a common identifier system.
- Deposit the built container image in a public container registry, such as Docker Hub, Quay.io or a publicly available and well supported institutional registry for container images.

### 10. Provide reproducible and documented builds

While containers strive to make research reproducible and transparent, it is equally essential that the process of creating and building containers themselves is transparent and reproducible. Many Docker containers do not provide an associated *Dockerfile*, which would allow an independent party to reproduce and verify the container build independently. Other build procedures rely on the presence of specific web resources, download binary files from the internet or can only be built with in-house resources that are not available to the public.

With BioConda and BioContainers every recipe is available and the mechanisms to create and build it. The Biocontainers registry provides a view to each recipe. Our recommendation is to provide clearly documented steps on how to generate all the binaries directly from the source code; if it is possible, engage with one of these two open-source communities to make your recipe available. Adding documentation to BioContainer and Conda recipes will allow the author as well as users to understand the build process and modify it their needs. If a particular resource may not be readily available or consists of a binary file, provide further instructions on how to re-create this resource (e.g. a link to a second recipe that creates the resource).

### 11. Provide helpful usage message

Usability and discoverability are crucial for packaged containers. If your tool provides a help “ *-h*”, “ *--help*” or “ *?*” message, consider providing this as the default command, “CMD” in case of a *Dockerfile*. If your tool does not provide a default usage message, consider providing this information in an ancillary “README.md” message. Your tool’s help or usage message is a useful place to provide a list of commands in logical groups, along with each command, giving a brief description, defaults, required arguments, and options.

## Conclusions

This manuscript promotes and encourages the adoption of package and container technologies to improve the quality and reusability of research software. The recommendations share a set of core views that are summarised below:

- *Simplicity*: the encapsulated software should not be a complex environment of dependencies, tools and scripts.
- *Maintainability*: the more software included in the container, the harder it is to maintain it, especially when the software comes from different sources.
- *Sustainability*: the developers of the software should be engaged or made aware of supporting the sustainability of the container.
- *Reusability*: a tool container should be safe to reuse by any other workflow component or task through its access interface.
- *Interoperability*: different tools should be easy to connect and exchange information.
- *User’s acceptability*: a tool container should perform a specific atomic task, so it is easier to check and use.
- *Size*: containers should be as small as possible. Smaller containers are much quicker to download and therefore they can be distributed to different machines much quicker.
- *Transparency*: containers should be transparent in how they are built, which tasks they are designed to perform and how the build process can be reproduced.

As with many tools, a learning curve lays ahead, but several basic yet powerful features are accessible even to the beginner and may be applied to many different use-cases. For users involved in scientific research and bioinformatics interested in this topic without experience working with software packages or containers, we recommend exploration and engagement with the BioContainers initiative.

## Data availability

No data is associated with this article.

## Supplementary material

**Supplementary File 1. Additional guidelines to make your container smaller.**
