# Supplementary material for: Recommendations for the packaging and containerizing of bioinformatics software
Source: F1000Res. 2019 Mar 20;7:ISCB Comm J-742. Originally published 2018 Jun 14. [Version 2] doi: 10.12688/f1000research.15140.2 (PMC6738188; doi:10.12688/f1000research.15140.2)
Supplement: Supplementary file 1 [file f1000research-7-20358-s0000.tgz › 6185852b-f373-4cf7-8977-ef150a57c0e0.docx]

**Supplementary Information: Recommendations to package and containerize bioinformatics software**

Bjorn Gruening ^2^, Olivier Sallou ^3^, Pablo Moreno ^1^, Felipe da Veiga Leprevost ^4^, Hervé Ménager ^5^, Dan Søndergaard ^6^, Hannes Röst ^7^, Timo Sachsenberg ^8^, Brian O'Connor ^9^, Fábio Madeira ^1^, Victoria Dominguez Del Angel ^10^, Michael R. Crusoe ^11^, Susheel Varma ^1^, Daniel Blankenberg ^12^, Rafael C Jimenez ^13^, BioContainers Community, Yasset Perez-Riverol ^1^

^1^ EMBL-European Bioinformatics Institute (EMBL-EBI), Hinxton, Cambridge, UK.

^2^ Bioinformatics Group, Department of Computer Science, University of Freiburg, 79110, Freiburg, Germany.

^3^ Institut de Recherche en Informatique et Systèmes Aléatoires (IRISA/INRIA) - GenOuest platform, Université de Rennes 1, Rennes, France.

^4^ Department of Pathology, University of Michigan, Ann Arbor, Michigan, USA.

^5^ Center of Bioinformatics, Biostatistics and Integrative Biology Institut Pasteur Paris, France.

^6^ Aarhus University, Bioinformatics Research Centre, C.F. Møllers Allé 8, Aarhus DK-8000, Denmark.

^7^ The Donnelly Centre, University of Toronto, 160 College Street, Toronto, Ontario M5S 3E1, Canada.

^8^ Universität Tübingen, Wilhelm Schickard Institut für Informatik, Applied Bioinformatics Group,D-72076 Tübingen, Germany.
^9^ Computational Genomics Lab, UC Santa Cruz Genomics Institute, University of California Santa Cruz, Santa Cruz, California, USA.

^10^ Institut Français de Bioinformatique (Elixir-FR), UMS3601-CNRS, Université Paris-Saclay, Orsay, 91403, France.

^11^ Microbiology and Molecular Genetics, Michigan State University, East Lansing, MI, USA.

^12^ Genomic Medicine Institute, Lerner Research Institute, Cleveland Clinic, Cleveland, OH, USA.

^13^ ELIXIR Hub, Cambridge, CB10 1SD, UK.

**1. Guidelines to make your container smaller**

**1.1 Multi-stage Dockerfile**

An example of a multi-stage build is provided in the following Dockerfile which uses a rather image (600 MB) large docker for building a go application while using a small (6 MB) distribution based on Alpine. In order to a more readable approach you can name your build stages. By default, the stages are not named, and you refer to them by their integer number, starting with 0 for the first FROM instruction. However, you can name your stages, by adding an as _<NAME>_ to the FROM instruction.

FROM golang:1.7.3 as builder
WORKDIR /go/src/github.com/alexellis/href-counter/
RUN wget https://raw.githubusercontent.com/go-training/helloworld/master/main.go
RUN mv main.go app.go
RUN CGO_ENABLED=0 GOOS=linux go build -a -installsuffix cgo -o app .

FROM alpine:latest
RUN apk --no-cache add ca-certificates
WORKDIR /root/
COPY --from=builder /go/src/github.com/alexellis/href-counter/app .
CMD ["./app"]
