# Supplementary material for: Recommendations for the packaging and containerizing of bioinformatics software
Source: F1000Res. 2019 Mar 20;7:ISCB Comm J-742. Originally published 2018 Jun 14. [Version 2] doi: 10.12688/f1000research.15140.2 (PMC6738188; doi:10.12688/f1000research.15140.2)
Supplement: Peer review history

# Peer review: Reviewer response for version 2

**DOI:** 10.5256/f1000research.20358.r53246

**Published:** 2019-09-09

**Authors:** Evan Floden

**Affiliations:** 1 Centre for Genomic Regulation (CRG), Barcelona Institute of Science and Technology (BIST), Barcelona, Spain

The article provides a series of best practise proposals on the important subject of encapsulating bioinformatics software. Conda and containers are fast becoming indispensable technologies for the deployment, sharing and reuse of bioinformatics tools and analyses. The availability of software through a Bioconda package or BioContainer is becoming a prerequisite for the adoption and use of any given bioinformatics tool. The widespread use of packages and containers has been made possible through the use of workflow managers such as Nextflow and Galaxy that wrap the standard commands into Docker or Singularity commands, mount the required volumes in the container or even build the Conda environment at run time. The recommendations provided in the article are sensible and well justified. The joint focus on both package managers and containerisation technologies allows the reader to compare and contrast the approaches with examples, whilst providing a description of shared resources such as Bioconda Packages being built as BioContainers.

 One comment I have relates to the “One tool, one container” recommendation. In reality, this is often not practical. For example, the inclusion of utility tools for the basic reading, writing and conversion of files (e.g. Samtools) often becomes a practical requirement. The author's state “is important to highlight that workflow composition is not addressed in the scope of this article as we are tied to the one container = one tool paradigm”, however, the use of containers without a workflow manager is not recommended for the majority of users. This recommendation could be updated to include for the recent multi-container registry.

 Minor fixes:

1. “Is important to highlight that” should be “It is important to highlight that”. 
2. “which will be the one user will download” should be “which will be the one users will download”.
3. Reference 16 (Tyler J, Frith GH: Primary drug abuse among women: a national study. Drug Alcohol Depend. 1981; 8(4): 279–86 ^1^) appears to be incorrect.

I confirm that I have read this submission and believe that I have an appropriate level of expertise to confirm that it is of an acceptable scientific standard.

## References

[1] : Primary drug abuse among women: a national study. *Drug Alcohol Depend*.1981;8(4) : 10.1016/0376-8716(81)90037-5 279-86 10.1016/0376-8716(81)90037-5 6978244

# Peer review: Reviewer response for version 2

**DOI:** 10.5256/f1000research.20358.r45998

**Published:** 2019-06-17

**Authors:** Ka Yee Yeung, Daniel Kristiyanto

**Affiliations:** 1 Institute of Technology, University of Washington Tacoma, Tacoma, WA, USA; 2 University of Washington, Seattle, WA, USA

In particular, the authors addressed the wording issues that we pointed out in the previous review. E.g. 1) Instead of claiming that "BioConda is the most popular package manager", the author has rephrased it into "Bioconda is a popular package manager". 2) They removed the recommendation to use alpine altogether to consistent with the sample docker. 

 We also tested the provided sample docker file. We were able to build the container and to run it successfully. A small issue is that it takes almost 20 Gb traffic to build the container, resulting in 1.3 Gb docker image --contradicting their recommendation to reduce container as small as possible.

We confirm that we have read this submission and believe that we have an appropriate level of expertise to confirm that it is of an acceptable scientific standard.

# Peer review: Reviewer response for version 1

**DOI:** 10.5256/f1000research.16494.r36273

**Published:** 2018-08-02

**Authors:** Monther Alhamdoosh

**Affiliations:** 1 CSL Limited, Parkville, Vic, Australia; 2 Bio21 Institute, University of Melbourne, Parkville, Vic, Australia

Gruening *et al.* addressed a very important issue in the field of bioinformatics, which is the reproducibility of research analytics. The authors clearly highlighted the importance of software packaging in containers in order to enable fast deployment and building of bioinformatics workflows and analytics. Namely, they focused on the importance of documenting software versions, the availability of source codes and the need to adopt a license when using third-party software.  Extending the definition of a software package to include command line tools, GUIs, APIs, scripts and specialized software packages is a very good idea although I have some reservations (see below). The authors proposed eleven recommendations to improve software packaging and containerization in bioinformatics. They also present some of the best practices as part of their recommendations. 

 I have a few comments on this manuscript that authors would probably like to address in their revised version. 

 Major comments

 ----------------------

- It would be helpful if you could provide a figure on how BioContainers, BioConda and Docker talk to each other in light of these recommendations. Probably, this figure from your GitHub can be adopted https://github.com/BioContainers/specs/blob/master/imgs//workflow.png
- It would be helpful to show an example of how to make use of these packaged and containerized objects. 
- Some weblinks need to be fixed on the BioContainers website, e.g., http://biocontainers.pro/docs/developer-manual/deploy-dockerhub/, to enable new users to adopt these recommendations. 

Minor comments

 ----------------------

- Bioconductor R packages usually released in versions following milestone releases of Bioconductor. Why are they distributed in separate containers? How do you make sure that people will not use different versions from different releases? 
- The use of the words "computational biology" and "bioinformatics" interchangeably is confusing. I would recommend using one of these two words.
- The purpose of the first recommendation is not clear. It might need to be pronounced further.
- The concepts of the metadata and tag are not explained clearly. This might make it difficult for people not familiar with containers to understand Recommendation 3. 

Overall, the manuscript introduces a great way of standardising bioinformatics research and empowering reproducibility of data analytics in the biomedical field.

I confirm that I have read this submission and believe that I have an appropriate level of expertise to confirm that it is of an acceptable scientific standard, however I have significant reservations, as outlined above.

# Response

**Published:** 2018-11-20

**Authors:** olivier sallou

**Affiliations:** University of Rennes 1, France

Hi, thanks for your comments.

 Regarding usage examples, we tried to avoid explaining Docker concepts as it does not focus on Docker only. Indeed containers will work with any container image compliant image (rkt, singularity), and would certainly need further explanations on what are Docker volumes etc.... We expect user to understand the basis of container usage.

 Regarding web links, you're right, some examples in documentation need to be fixed

 About R packages, they could be in a single container, but this would create a huge container to manipulate and difficult to maintain (and which packages should be put?). If user really needs a container with multiple R packages, he can create one based on an existing container and add what is necessary. We focus on programs (R for example) and not libraries (r-xxx lib) as it is not possible to provide a container matching all user needs. Some libraries are provided either with automatic package injection from other sources or for the need of a few users.

 If a user need a workflow/composition of multiple *tools*, user should use scripting of container enabled workflow tools that will execute one task = one tool = one container, and avoid putting all workflow tool in a single container.

# Peer review: Reviewer response for version 1

**DOI:** 10.5256/f1000research.16494.r35062

**Published:** 2018-07-16

**Authors:** Ka Yee Yeung, Daniel Kristiyanto

**Affiliations:** 1 Institute of Technology, University of Washington Tacoma, Tacoma, WA, USA; 2 University of Washington, Seattle, WA, USA

The authors addressed the reproducibility issue in bioinformatics research. While many factors can be attributed to this issue, the paper focused on the tools and software packages. With more research being done and more tools in Bioinformatics made available as packages and complex workflows, this work is a good reminder of the importance of writing well-written products that the community can use and replicate.

 The manuscript provided sound advice in building and maintaining these tools. It also presented easy to follow sample and template of the solutions. The guidelines are itemized with a clear explanation of the necessity of the rules. Not only for bioinformatics community, but the guidelines are also general enough to serve as good practices for general use in writing software packages. Recommendations include choosing a cross-platform package manager, encapsulating a single tool in each container, reducing the package size, keeping data outside the container, providing testing logic, checking licensing issues, and providing usage messages.

 As a demonstration, the authors scoped the samples and templates to BioConda and BioContainer, with claims that these software packages are the most popular among bioinformatics community.  The reviewers would like to request a reference for the statement “Conda, the most popular package manager in research software”. The example is concise and easy to follow. However, the sample does not strictly reflect what the guidelines proposed in the manuscript. For instance, the manuscript recommended the readers to use Alpine for the base image for Docker containers, the example template in “Box 2” used another pre-built Docker Image instead.  The reviewers would like to request the authors to explain or revise the example.

 Additionally, the manuscript suggested to break down each package or containers as an atomic task to enhance modularity in Guideline #2. However, the authors did not discuss the crucial issue of how to assemble these different packages into a single workflow. Especially, as the authors also noted, bioinformatics workflows often consist of complex pipelines. Coverage on how parameters should be organized would also be useful (e.g., using environment variables versus a text file or command line parameters).

 In addition, recommendation #4 (avoid entry point) assumed that the tools would always be command-line based; this may not always be the case. Please elaborate when handling GUI based containerized tools.

 Lastly, we failed to replicate the provided sample script for Comet software with error `Syntax error - can't find = in "open". Must be of the form: name=value` on Docker for Mac: Version 18.03.1-ce-mac65 (24312).  Please double check.

We confirm that we have read this submission and believe that we have an appropriate level of expertise to confirm that it is of an acceptable scientific standard, however we have significant reservations, as outlined above.

# Response

**Published:** 2018-11-20

**Authors:** olivier sallou

**Affiliations:** University of Rennes 1, France

Hi,

 regarding cCnda reference as "the most popular", I agree the should be rephrased as "a popular"

 Regarding Alpine base image, you suppose you refer to "Use a lightweight base image for your deployed container, such as Alpine. Only use a more mainstream image such as Ubuntu or CentOS if absolutely required for your application to run."

 Biocontainers (Dockerfile based) makes indeed use of an other image (Ubuntu based) to allow user to exec bash/vim/... operations in containers and easily extend them. If not needed (oout of biocontainers scope), Alpine is recommended to limit container size and scope. This sentence should be rephrased to explain this difference.

 Workflow composition is not addressed in the scope of this article as we are tied to the one container = one tool paradigm (though not being too strict on this...)

 Workflow tools (nextflow, cwl based or others) will execute 1 task per tool, each tool being one container. But maybe that, indeed, it would be worth to explain users the such tools are needed for workflow execution in containers context.

 Recommendation #4 about entry point still applies for GUI based tools. A GUI tool is just a cmd line tool that "pops up" a frontend. Only difference is the need to mount some x11 directorties with hosts  for Docker but Docker usage/tutorial is out of the scope of this article.

 About Comet example, there are some quotes errors in text (text editor related) and a few missing space. We will fix this, thanks for pointing to that error
